# Supplementary material for: An ANI gap within bacterial species that advances the definitions of intra-species units
Source: mBio. 2023 Dec 12;15(1):e02696-23. doi: 10.1128/mbio.02696-23 (PMC10790751; doi:10.1128/mbio.02696-23)
Supplement: Supplemental Material — Supplemental methods, figures, and Tables. [file mbio.02696-23-s0001.pdf]

An ANI gap within bacterial species that advances the definitions of intra-species units.

Luis M. Rodriguez-R[1*], Roth E. Conrad[2,*], Tomeu Viver[3], Dorian J. Feistel[2], Blake G. Lindner[2], , Stephanus N. Venter[4], Luis Orellana[5], Rudolf Amann[5], Ramon Rossello-Mora[3], and Konstantinos T. Konstantinidis[2]

SUPPLEMENTARY METHODS AND RESULTS.

Statistically testing the presence of an 99.2-99.8% ANI gap.

1. Bootstrapped peak finding on kernel density estimates to estimate variation and confidence intervals of gaps (or valleys) in the ANI distribution. We performed a bootstrap resampling analysis to produce estimates and confidence intervals for local minimum and maximum ranges in the ANI distribution. We wrote custom Python code for this task that utilized functions from the NumPy, Pandas, SciPy, Matplotlib, and Seaborn packages (1-5). First, we filtered our species collection to select only species with at least 100 pairwise genome comparisons with ≥ 96.5% ANI leaving 154 species for the bootstrap analysis. Next, for each bootstrap iteration, we randomly sampled with replacement the ANI of 100 pairwise genome comparisons for each species, computed the kernel density estimate across the combined distribution for all 154 species (scipy.stats.gaussian_kde, bw_method=0.15), and identified local minimums and maximums (scipy.signal.find_peaks, default settings). We repeated this for 10,000 bootstrap iterations. Figure S8B shows the results of a single bootstrap iteration and Figure S8C show the results from all 10,000 bootstrap iterations.

Single species ANI distributions grouped into four major patterns based on kernel density estimates. We selected species with at least 45 pairwise genome comparisons (ANI estimates) ≥ 95% ANI (i.e., 10 x 10, choose r=2 genome combinations). We computed kernel density estimates using the full ANI distribution for each species (scipy.stats.gaussian_kde, bw_method=0.15), and we identified local minimums and maximums for each species (scipy.signal.find_peaks, default settings). We then grouped the species into four groups as follows: Group1 was defined as species found to have a local minimum (valley) in the [99.2-99.8] % ANI range (majority of species), Group 2 was defined as species with mean ANI estimates ≥ 99.5% (highly clonal), Group 3 was defined as species without a local minimum in the [99.2-99.8] % ANI range (local minimum is shifted and deviates from the majority group 1 pattern), and Group 4 is defined as species with a local maximum in [99.2-99.8] % ANI range rather than a minimum. Finally, we computed histogram counts with bin size of 0.1% ANI for each species, normalized by species total ANI comparisons (counts) and multiplied by 100 so each bin count shows the percent of total. We produced heatmaps for each group displaying the histogram bin count percentage where each species is a row, and each cell is the percent of the row (species) total.

The code and figures have been placed in the 03_Bootstrap_analysis directory of the GitHub repository for this manuscript.

2. Statistical testing of the presence of an ANI distribution gap using dMA's *B,* Hartigan's dip test, and Sarle's B bimodality score.

For each tested species, we applied three tests to detect multimodality of the ANI distribution. First, we used dMA's *B* statistic (6), defined as:

$$B = \left| \mu - \frac{\sum_{i=1}^{L} m_i x_i}{\sum_{i=1}^{L} m_i} \right|$$

Where μ is the sample mean, and the second term is defined by a series of L bins, for which the i-th bin has midpoint x_i and a total of m_i observations. Bin sizes were set as 0.1 units of ANI (0.1%). This method was reimplemented here in the *R* programming language v4.3.1. Next, we used Hartigan's dip test (7) as implemented by the diptest package v0.76.0 for the *R* programming language. We applied a p-value adjustment for multiple testing using the false discovery rate method (8) as implemented in the p.adjust function with method = "fdr" from the R programming language base package stats v4.3.1. Finally, we also implemented Sarle's *B* bimodality score, defined as:

$$B = \frac{{m_3}^2 + 1}{m_4 + \frac{3(n-1)^2}{(n-2)(n-3)}}$$

Where n is the sample size, and m_3 and m_4 are the unbiased estimators of skewness and kurtosis, respectively. We estimated these values using the implementations in the e1071 package v1.7.13 for the R programming language using the parameter type = 2 to ensure unbiasedness.

Statistical testing of the prevalence of 99.5% as central value of the ANI gap

For each tested species, we produced smooth density curves of ANI using the density function from the base package stats v4.3.1 for the R programming language, using a fix maximum value of 100% and a bandwidth of 0.1% ANI. Density curves were truncated when extending below 96%.

For each curve, we applied the peak picker algorithm of Weber et al (9) as implemented in the pickPeak package v0.11 for the *R* programming language. The peak picker was applied to the negative of the smooth density curves in order to find valleys (instead of peaks). Since some distributions resulted in the detection of multiple valleys, only one valley was selected at random per species in order to equalize the importance of all species without introducing any biases. Next, the original ANI values (without smoothing) for each species were clustered into two groups (G = 2) using parametrized finite Gaussian mixture models as implemented by the mclust package v6.0.0 (10) with ANI as the single input variable. The middle point between them was calculated as the median of the four endpoints (two per group), and used as cut points separating the two detected data populations. Finally, the Otsu thresholding method, derived from data processing, was used to detect most-discriminant thresholds in each species (11). We reimplemented Otsu's criterion in the *R* programming language, with the criterion being defined as:

$$C(t) = \frac{n_1 \sigma_1^2 + n_2 \sigma_2^2}{n \sigma^2}$$

Where n_i and σ_i^2 are the size and variance of the *i*-th group, respectively, and *n* and σ^2 are the size and variance of the entire set, with groups being defined as *i=1* for ANI values below a given threshold *t* and *i=2* for values greater than or equal to *t*. We estimated the threshold *t* that minimized *C(t)* between 96% and 100% ANI every 0.05%, and broke ties at random.

The distribution of detected valleys, cut points, and thresholds were visually inspected using both histograms and smoothed density curves, and for each case the most likely value (mode) was estimated using the meanshift mode estimator (12), as implemented in the modeest package v2.4.0 for the *R* programming language.

Results:

In order to systematically evaluate the presence of the ANI gap around 99.5% across species, we used the collection of 330 species described above to test two aspects of these distributions: **(1)** How frequently do we observe a distribution of ANI values with more than one peak? And **(2)** when two or more peaks are detected, how often they separate around 99.5% ANI? The ANI values within each species were filtered to remove self-comparisons as well as values below 96%, and only species with 5 or more remaining ANI values were retained (n = 314).

To answer the first question, on the prevalence of a valley (regardless of value), we used three alternative approaches. First, we first calculated a recently introduced index of bimodality we'll term here dMA's *B* (6). All the 314 ANI distributions resulted in values below 0.04, so none was detected as bimodal when compared against the author's proposed cutoff of 0.1. However, we noticed that distributions with a majority of empty bins consistently resulted in low dMA's *B* values, even from clearly bimodal (or multimodal) distributions, which we confirmed using simulations (data not shown). Indeed, recalculating the scores with a bin size of 1 unit of ANI resulted 26.4% of the distributions being detected as bimodal. However, since our main interest here is on distinguishing peaks below and above 99.5%, this method simply cannot provide sufficient resolution. Additionally, we found a severe sensibility to the bin size parameter (62.4% detected as bimodal with bin size 2, and 75.4% with bin size 5) and therefore don't consider this method further.

As a second approach, we applied Hartigan's dip test (7), with a significance value of 95%. This resulted in 74.2% of the tested species being detected as significantly multimodal (p-value < 0.05), or 72% when adjusting p-values for multiple testing. Finally, we applied a traditional contrast of the kurtosis and the square of the skewness (13) in the form of Sarle's *B* score. This value was contrasted against the critical value of 5/9 for normal and exponential distributions (14), resulting in 62.1% of the ANI distributions being detected as multimodal. We used Sarle's *B* method to define multimodal distributions for subsequent analyses. However, we repeated all subsequent analyses using Hartigan's dip test alone, as well as defining any distribution detected by either method as multimodal, and obtained nearly identical results.

To answer the second question, on the prevalence of 99.5% as the typical gap given multimodality, we applied four methodologies. First, we produced smooth distributions of ANI in the range between 96 and 100% for each studied species, and inspected visually these

distributions. The pile-up of the resulting distributions revealed a strong gap around 99.6% ANI, both when including only multimodal distributions or all distributions (Fig. S9A). This was consistent with our previous observations on individual species and on summary distributions across species. Next, we adapted a peak picker algorithm to the detection of valleys by applying it to the negative of the smoothed densities (9). The distribution of detected valleys was largely accumulated around the proposed 99.5% ANI value, with a most likely value (mode) estimated at ANI 99.526% (Fig. S9 B). This method appeared to be impervious to the definition of multimodality, with similar distributions for multimodal, unimodal, and combined sets of species. This detection of valleys near 99.5% even in species that were not identified as multimodal possibly indicates false positives, presumably due to the sharp decrease below 100% ANI. Although visual inspection of the smoothed densities indicated that the majority of valleys were properly detected (compare Fig. S9A and B), two independent methods that don't rely directly on distribution shape were also applied.

First, ANI values (without smoothing) were classified in one of two Gaussian distributions using model-based clustering, thereby forcing the detection of only the most prominent gap. To characterize the location of the gap, the middle point between the highest ANI from the lower group and the lowest ANI from the higher group was calculated, and set as the cut point between the two Gaussian components. The histogram of cut points, their smoothed density, and most likely value were all estimated as described for valleys above (Fig. S9 C). The most likely value was estimated at 99.555% ANI. Notably, the cut points estimated from non-multimodal species displayed a flat distribution without preference for values near 99.5%, indicating that false positive gap detections were uniform and confirming that the accumulation of values around 99.5% ANI was not an artifact of the 100% density spike. Finally, a method for the detection of thresholds derived from image processing was applied on the ANI values, which relies on identifying the threshold that minimizes Otsu's criterion (11). Again, the histogram, smoothed density, and most likely value of the distribution of detected thresholds were estimated (Fig. S9 D). The most likely value was estimated at ANI 99.453%, while values around 99.5% appeared at low frequencies in non-multimodal species, indicating that no effect from false detection was at play. It's worth noting that Otsu's criterion may favor central points for flat distributions, and therefore might unfairly disadvantage the detection of gaps near the edge of the distribution (such as 99.5% ANI). This effect can be seen on the high concentration of thresholds near 98% ANI (likely spurious), but even with this disadvantage the concentration of thresholds around 99.5% ANI was robustly recovered.

Importantly, in all the above analyses species were treated equally, irrespective of data set size. In order to evaluate if any of the above metrics were biased by data size, we compared the squared root of the number of ANI values (roughly corresponding to the total number of genomes considered) with each of the above metrics (Fig. S9 E-F). No significant correlations were detected between the size of the data collection for each species and their corresponding values of bimodality (Pearson's R = -0.1, p-value = 0.075) or their detected ANI gaps with any of the methods above ($|R| < 0.053$, p-values > 0.35). All these results are shown in Figure S9.

SUPPLEMENTARY FIGURES AND TABLES.

Supplementary Table S1. Statistics of ST separation at different thresholds of ANI for *E. coli* closed genomes. The first header row indicates the different thresholds evaluated to distinguish genomes assigned to STs based on ANI. True Positives (TP) were defined as genome pairs with ANI >= threshold and the genomes were assigned to the same ST; True Negatives (TN) when the genome pair had ANI < threshold and the genomes were assigned to different STs; False Positives (FP) when the genome pair had ANI < threshold and genomes were assigned to the same STs; and False Negatives (FN) when the genome pair had ANI >= threshold and genomes were assigned to different STs. Precision, Recall, Accuracy, and F1 score were defined as shown on the Table (bottom).

Species	ST	Genomes	Datapoints	ANI >= 99.0				ANI >= 99.2				ANI >= 99.5				ANI >= 99.8			
				Precision	Recall	F1	Accuracy	Precision	Recall	F1	Accuracy	Precision	Recall	F1	Accuracy	Precision	Recall	F1	Accuracy
Escherichia coli	ST-10	228	77850	1	0.3318	0.4983	0.3318	0.9636	0.4678	0.6298	0.6242	0.5947	0.78	0.6749	0.8099	0.4452	0.7914	0.5698	0.777
Escherichia coli	ST-11	218	26498	1	0.8926	0.9433	0.8926	1	0.893	0.9435	0.893	1	0.9524	0.9756	0.9554	0.9679	0.9817	0.9748	0.9552
Escherichia coli	ST-131	104	5953	1	0.8997	0.9472	0.8997	0.9989	0.9446	0.971	0.9462	0.744	0.9686	0.8416	0.748	0.5429	0.9959	0.7027	0.5868
Escherichia coli	ST-167	65	23101	1	0.09	0.1651	0.09	0.9793	0.1144	0.2049	0.3155	0.8433	0.8421	0.8427	0.9716	0.2221	0.9978	0.3633	0.9299
Escherichia coli	ST-38	61	2372	1	0.7715	0.871	0.7715	0.9995	0.8539	0.921	0.8676	0.7005	0.8745	0.7779	0.6914	0.1055	0.8773	0.1883	0.2985
Escherichia coli	ST-410	59	3348	1	0.5111	0.6765	0.5111	1	0.5592	0.7173	0.5971	0.9819	0.7483	0.8493	0.822	0.3472	0.9917	0.5143	0.6649
Escherichia coli	ST-95	42	1213	1	0.7098	0.8303	0.7098	1	0.8367	0.9111	0.8615	0.9048	0.8422	0.8724	0.812	0.151	0.7558	0.2517	0.3627
Escherichia coli	ST-93	38	3256	1	0.2159	0.3551	0.2159	1	0.8155	0.8984	0.9512	0.8634	0.9019	0.8822	0.9502	0.5718	1	0.7276	0.9076
Escherichia coli	ST-405	35	630	1	0.9444	0.9714	0.9444	1	0.9444	0.9714	0.9444	0.995	0.9442	0.9689	0.9397	0.1849	1	0.3121	0.2302
Escherichia coli	ST-73	32	705	1	0.7035	0.8259	0.7035	1	0.8378	0.9117	0.8638	1	0.8378	0.9117	0.8638	0.3589	0.9271	0.5175	0.5291
Escherichia coli	ST-48	32	9648	1	0.0404	0.0777	0.0404	0.659	0.1101	0.1887	0.7708	0.0436	0.85	0.0829	0.961	0.0154	1	0.0303	0.9602
Escherichia coli	ST-69	29	457	1	0.8884	0.9409	0.8884	1	0.9927	0.9963	0.9934	0.6552	1	0.7917	0.6937	0.0665	1	0.1247	0.1707
Escherichia coli	ST-127	22	327	1	0.7064	0.8279	0.7064	1	0.913	0.9545	0.9327	1	1	1	1	0.645	1	0.7842	0.7492
Escherichia coli	ST-648	20	304	1	0.625	0.7692	0.625	0.9105	0.7588	0.8278	0.7632	0.6684	0.7744	0.7175	0.6711	0.0316	0.6667	0.0603	0.3849

Precision = TP / (TP + FP)
Recall = TP / (TP + FN)
F1 score = (2 * Precision * Recall) / (Precision + Recall)
Accuracy = (TP + TNP) / (TP + TN + FP + FN)

Supplementary Figure S1. Examples of individual species showing the 99.5% ANI gap (top) and species that deviate from this pattern (bottom). The figure is similar to Fig. 1 but only shows datapoints from individual species, one species per panel (see panel title). Note the multiple ANI gaps (or valleys) that are often observed for most species, and the consistent 99.5% ANI gap across species, with the possible exception of *Riemerella anatipestifer* and *Bordetella bronchiseptica*, where the gap may be shifted at around 99% ANI (*R. anatipestifer*) or not exist (*B. bronchiseptica*), respectively.

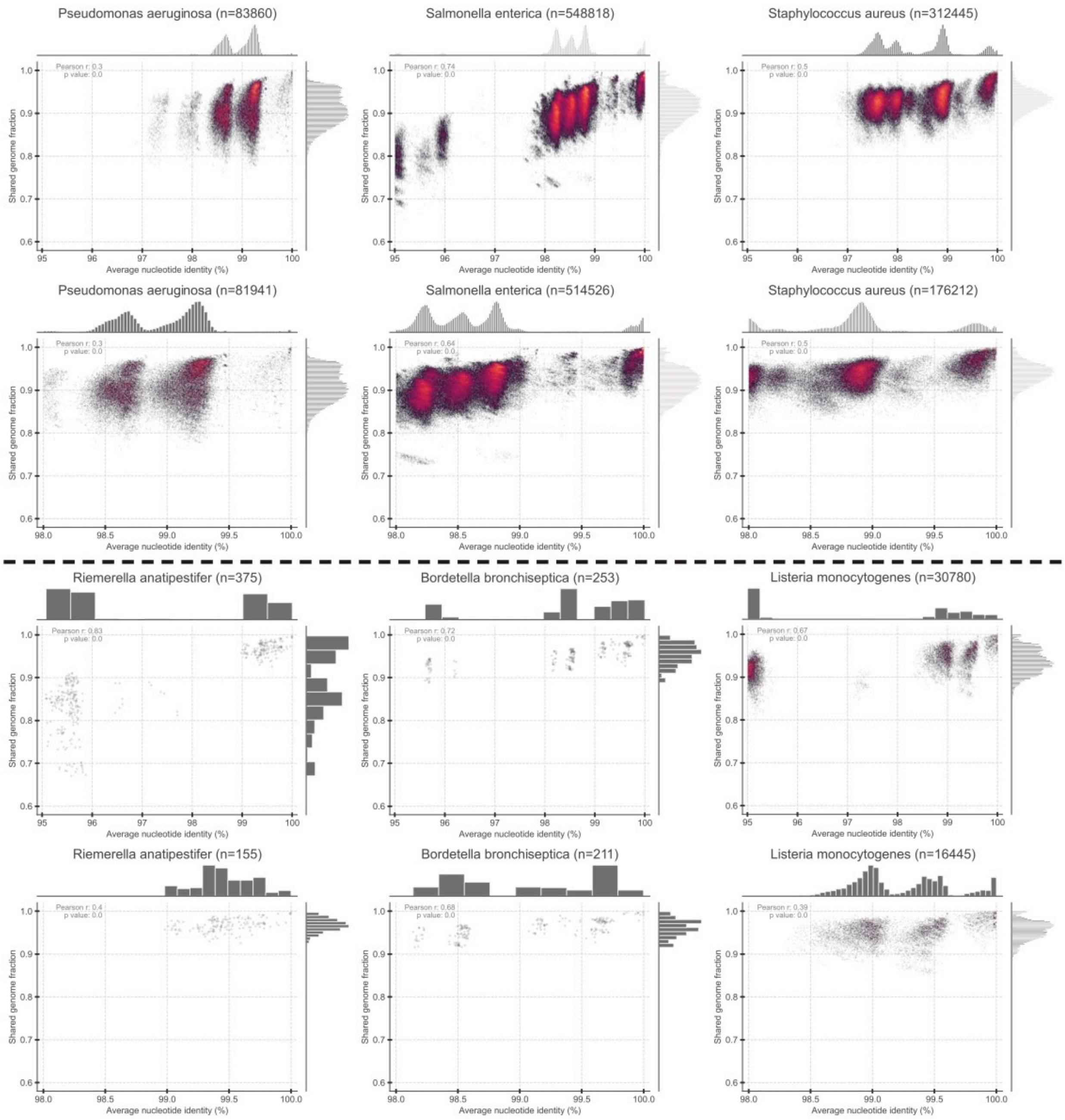

Supplementary Figure S2. Statistical support for the ANI gap observed based on all species used in this study. Species with at least 45 pairwise genome comparisons (ANI values) with ≥ 95% ANI were used for this analysis. We computed kernel density estimates using the full ANI distribution for each species (scipy.stats.gaussian_kde, bw_method=0.15), and we identified local minimums and maximums for each species (scipy.signal.find_peaks, default settings). We then grouped the species into four groups as follows: Group1 was defined as species found to have a local minimum (valley) in the [99.2-99.8] % ANI range (majority of species; Panel A; green box), Group 2 was defined as species with mean ANI estimates ≥ 99.5% (too clonal to call or the ANI gap exist but it is much broader and extends below 99% ANI; Panel B), Group 3 was defined as species without a local minimum in the [99.2-99.8] % ANI range but the local minimum is shifted compared to the Group 1 pattern or extents to <99% ANI (Panel C; e.g., *Aeromonas salmonicida* and *Yersinia enterocolitica*), and Group 4 is defined as species with a local maximum in the [99.2-99.8] % ANI range rather than a minimum (Panel D, e.g., *Riemerella anatipestifer*). Finally, we computed histogram counts with bin size of 0.1% ANI for each species, normalized by the species total number of ANI comparisons (counts) and multiplied by 100, so each bin shows the percent of total ANI values that fall in the bin. We produced heatmaps for each group displaying the histogram bin count percentage for each species in a separate row (species name is shown on the right). Note that this is an automatic (no human interference) approach in calling the ANI gaps and shows that the major gap is indeed the 99.2-99.8% ANI mentioned in the main txt. Further, notice that even in the Group 4 that includes the exceptions to the gap, several species might actually be consistent with the gap but they probably do not have enough genomes available yet to be assigned to Group 1 (e.g., *Acinetobacter baumannii*, *Rhizobium phaseoli*); the true exceptions to the main pattern appear to be rather infrequent overall.

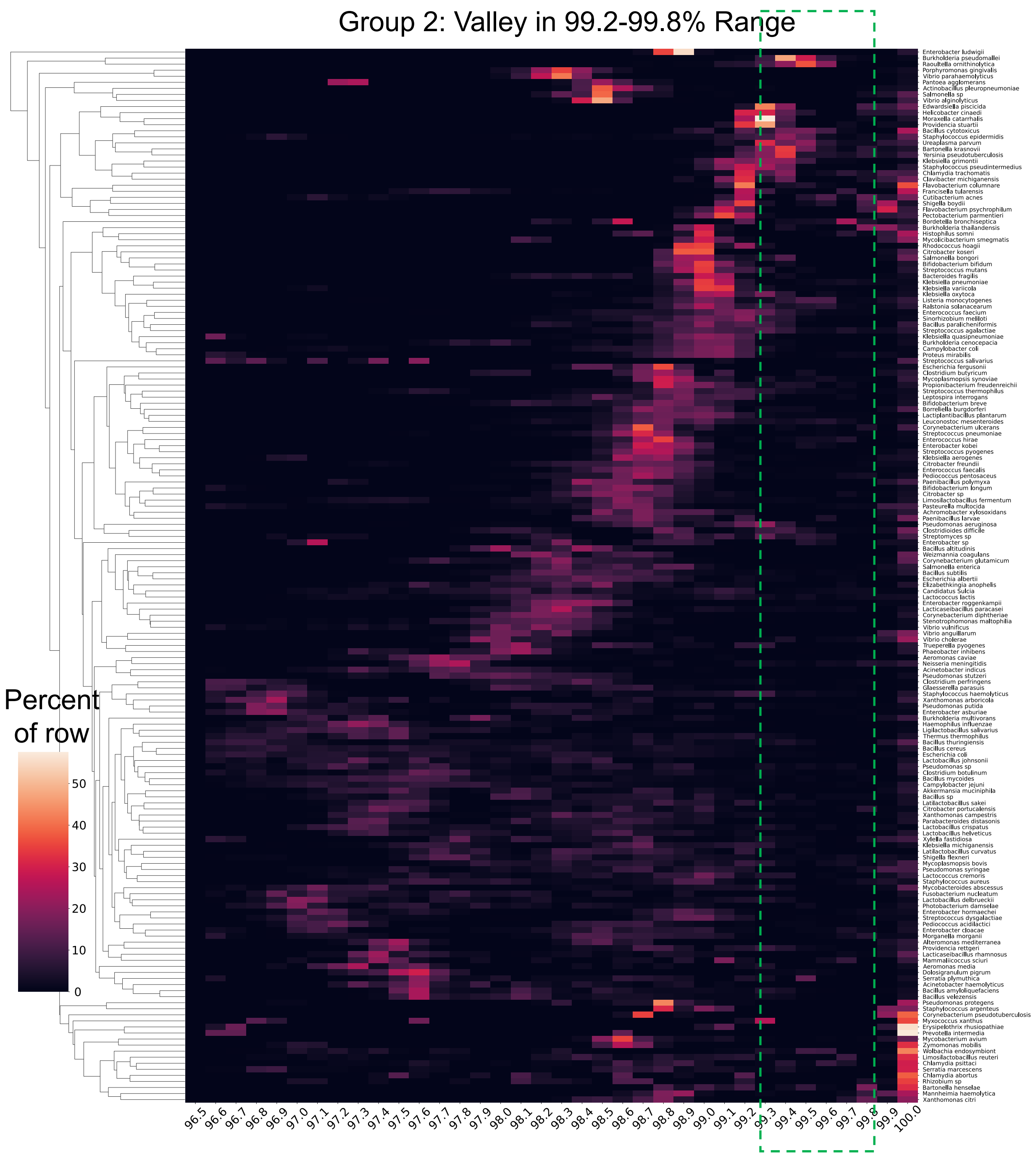

Panel A: Species with a clear 99.2-99.8% ANI gap

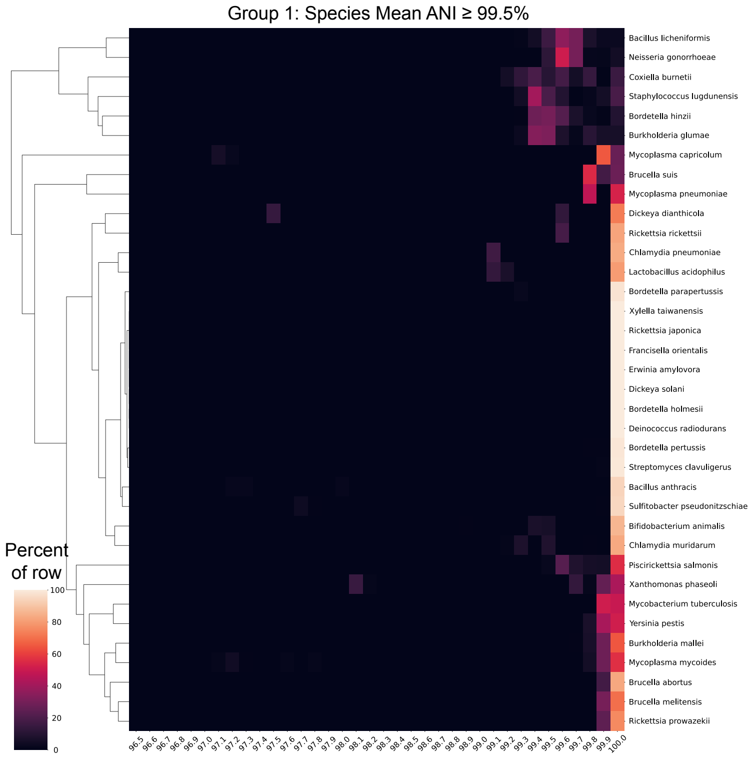

Panel B: Species too clonal to call (or the ANI gap extents below 99% ANI).

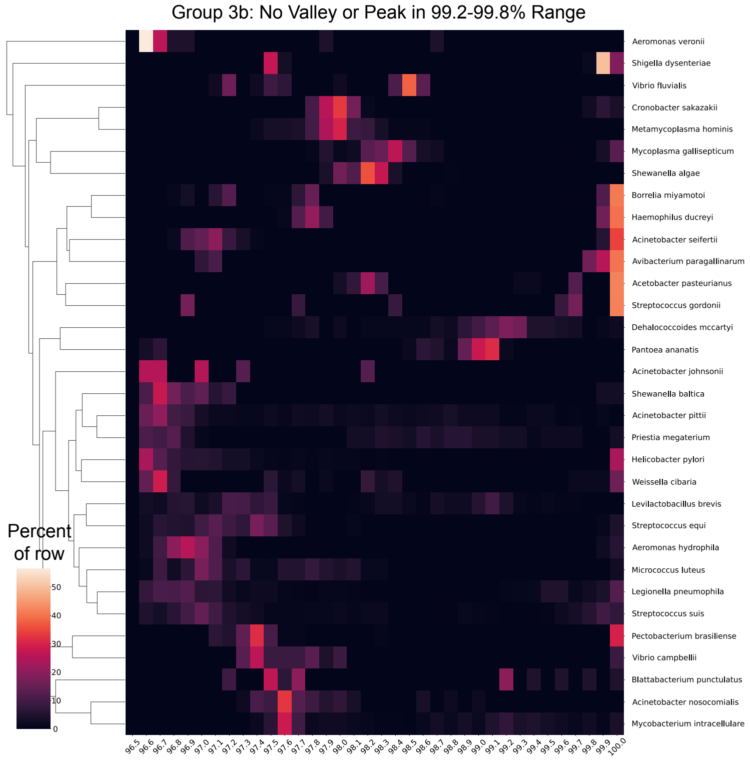

Panel C: Species with a clear ANI gap but shifted compared to the major 99.2-99.8% ANI gap.

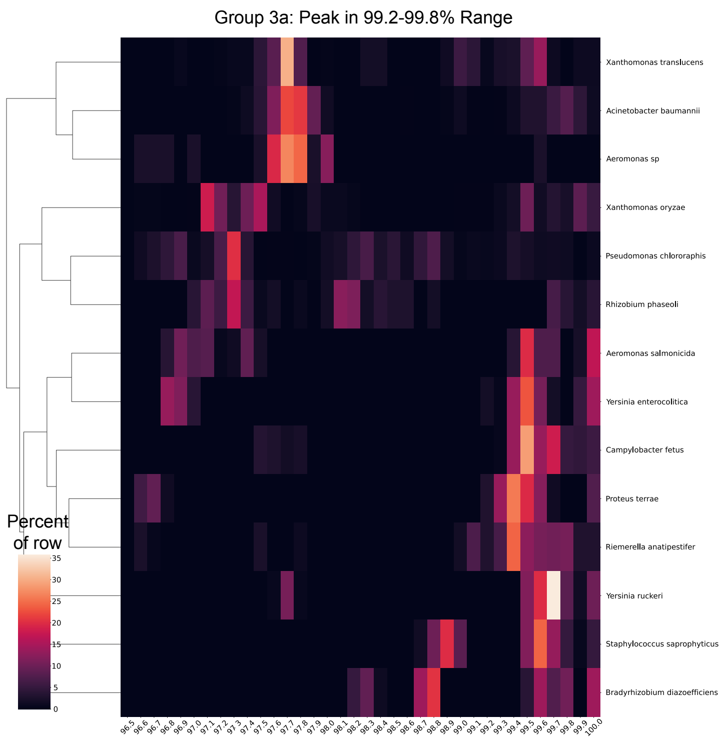

Panel D: Species with a clear maximum in the 99.2-99.8% ANI range.

Supplementary Figure S3. Clinical and environmental genomes show similar intra-species ANI value distribution patterns. The Fig. S3 is identical to Fig. 1 except that the 17,283 NCBI genomes were separated in **clinical** (n = 224 species; 27150 genomes; 2585561 genomes pairs with >95% ANI), for those associated with human or animal hosts (top), and **environmental** (n = 113 species; 3706 genomes; 61212 genomes pairs with >95% ANI), for those associated with biotechnological applications and/or no-host (bottom), based on their NCBI record. Note that while there are fewer environmental genomes overall, these do show the ANI gap around 99.5-99.9%, albeit a bit narrower compared to the clinical species (see text for additional discussion of this result).

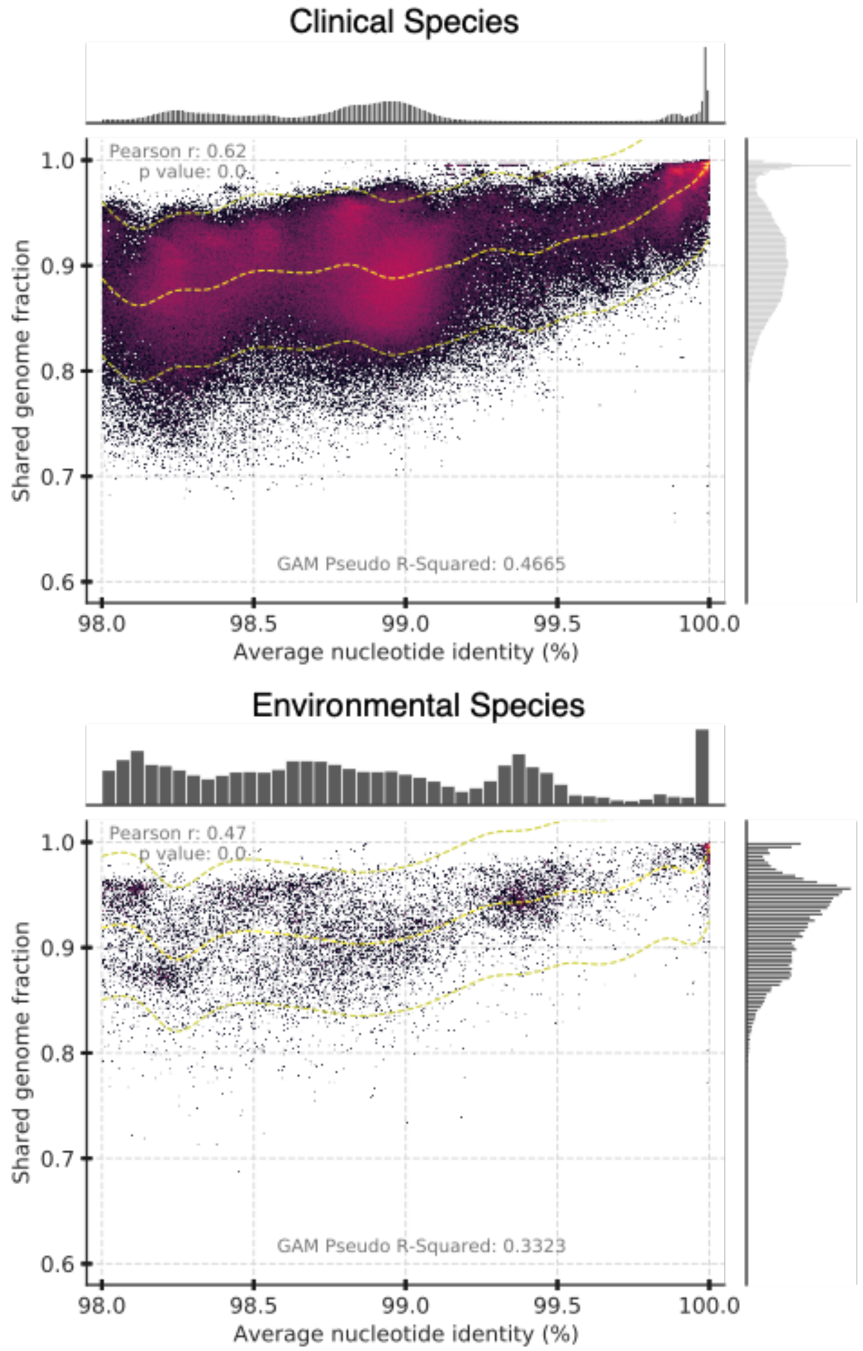

Supplementary Figure S4. Random subsampling of the genomes supports the 99.5% ANI gap. The figure is similar to Fig. 1 but only includes 256 (top Panel, 256000 genome pairs with >95% ANI) and 215 (bottom Panel, 215000 genome pairs with >98% ANI) species with exactly 45 datapoints (genome pairs; from all vs. all comparisons of 10 genomes) each. Note that the 99.5% ANI gap is apparent, albeit not as pronounced as in Fig. 1 due to the subsampling of the datapoints. That is, there are many more genome pairs showing <99% ANI than pairs showing >99% ANI, and thus the former pairs are represented much more frequently when restricting the analysis to 10 randomly-drawn genomes per species.

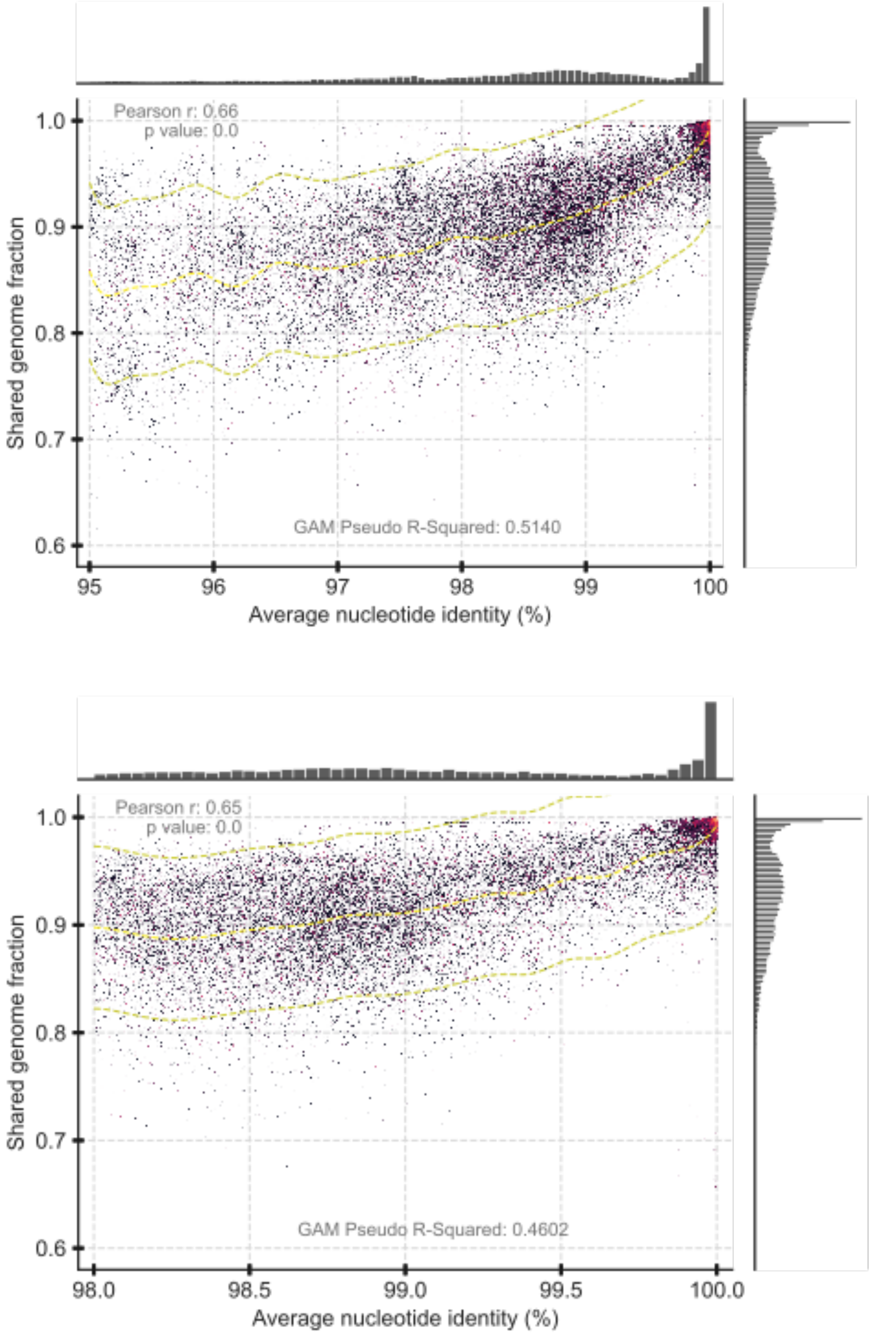

Supplementary Figure S5. The intra-species ANI gap is often present in natural populations recovered by long-read PacBio metagenomic sequencing from the surface waters of the North Sea. The underlying dataset is a PacBio HiFi long-read metagenome from surface waters of the North Sea after a seasonal algal bloom described previously (15). The graphs show the nucleotide (nt) identity distribution of individual reads against a reference MAG recovered from the same dataset. All reads shared at least 5Kbp of their >10Kbp length with the MAG for stringent results while similar distributions were obtained when reads were searched against themselves (data not shown). Top graphs show all reads sharing nt identity >70%; bottom graphs show the subset of these reads that share nt identity >98%. The reads represent 20 populations, which were adequately sampled (abundant), combined together (Panel A; n = 411790 reads), a single representative population of these 20 abundant populations that appears to be too clonal to assess the intraspecies gap, e.g., most reads showing nt identity >99% (Panel B; n = 23109), and another single population (of the 20 abundant populations) that, while the ANI gap is not clear when all reads belonging to the population were assessed (Panel C; 16817 reads), the gap becomes evident, albeit not as pronounced as those observed of other species (e.g., Figs. 2 and 3), when the analysis is limited to the rpoB-carrying reads (Panel D; 50 reads). Gap in Panel D refers to the lack of reads between 99.5% and 99.8% nt identity relative to <99.5% and >=99.8% (note that while the number of reads available -or coverage- is not high enough for the robust elucidation of the gap, the identity distribution in panel D appears quite different from that observed in Panel C, and consistent with the 99.5% ANI gap observed in other species). MAG A represents a novel genus of the *Flavobacteriaceae* family and MAG B a new member of *Schleiferiaceae* affiliated at the species level with GTDB's taxon s__UBA10364.

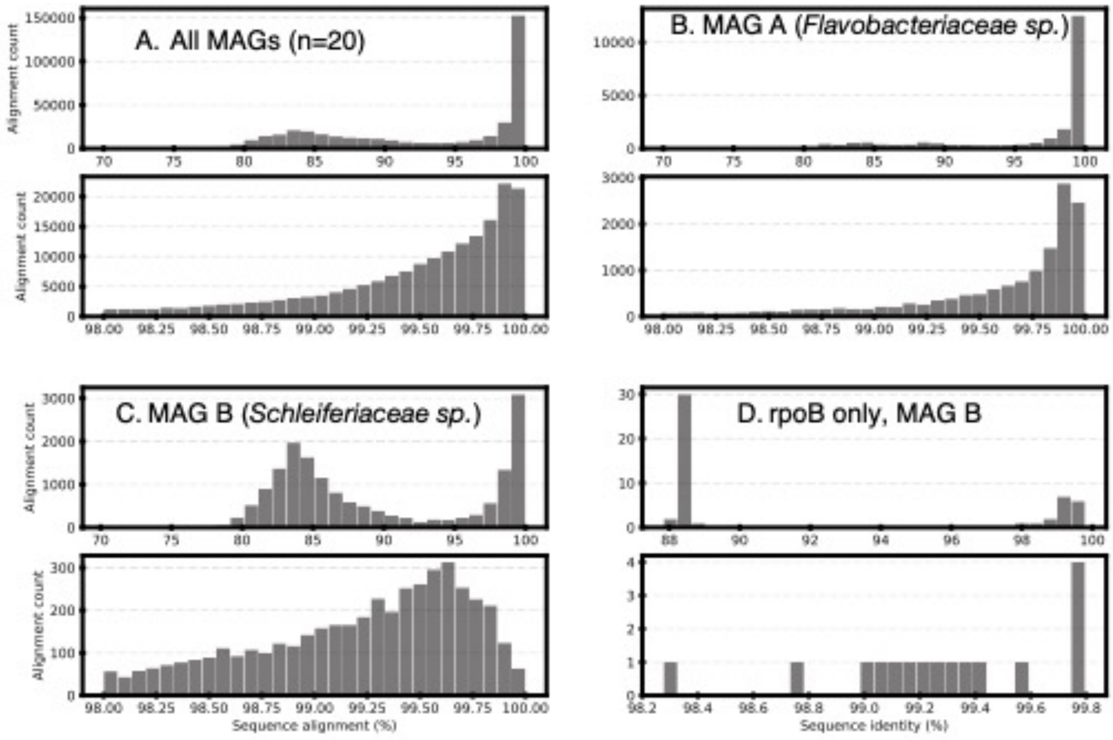

Supplementary Figure S6. Enrichment of hypothetical and mobile genes among the genes that differ between more closely related genomes. For this figure, we selected genes that are not shared between two genomes and recorded the category of the gene's annotation (legend) and the ANI value of the two genomes considered (x-axis). The fraction of the total genes that differed (not shared) between any genome pair comparison in the corresponding ANI bin (x-axis) and fall within the annotation categories is shown on the y-axis. For instance, 42% of genes not shared between two genomes with ANI values in the range of 99.2-99.8% were found to be Hypothetical. The percent listed at the top of each bin is the fraction of all genes that differed between any two genome pairs compared that fall into that bin (e.g., 67.58% of genes not shared between two genomes fall into the 98-99.2% ANI range). Genes were predicted using Prodigal with default settings for 16718 genomes from 279 species (40). For each species, genes were clustered with MMseqs2 at 90% amino acid sequence identity using the following parameters "--min-seq-id 0.90 --cov-mode 1 -c 0.5 --cluster-mode 2 --cluster-reassign". For each species, a binary matrix was generated from MMSeqs2 output with genomes as columns and gene clusters as rows, with the number one to denote a gene cluster presented in the corresponding genome. Gene differences were tabulated for genome pairs from this matrix and assigned to the corresponding ANI bin. Representatives of each gene cluster were annotated with EggNog Mapper using default settings. The Mobile category was generated using a keyword search of the annotation column using the keywords: transposase, phage, integrase, viral, plasmid, integron, and transposon. The Metabolism category was created using the assignments from the COG_cat column: C, G, E, F, H, I, P and Q. The COG_cat value of S was reported as Conserved Hypothetical. Any gene not receiving an annotation is reported as Hypothetical. All other gene assignments were reported as Other. Note the lower fraction of metabolic genes and, conversely, the higher fraction of mobile and hypothetical genes among more closely related genomes.

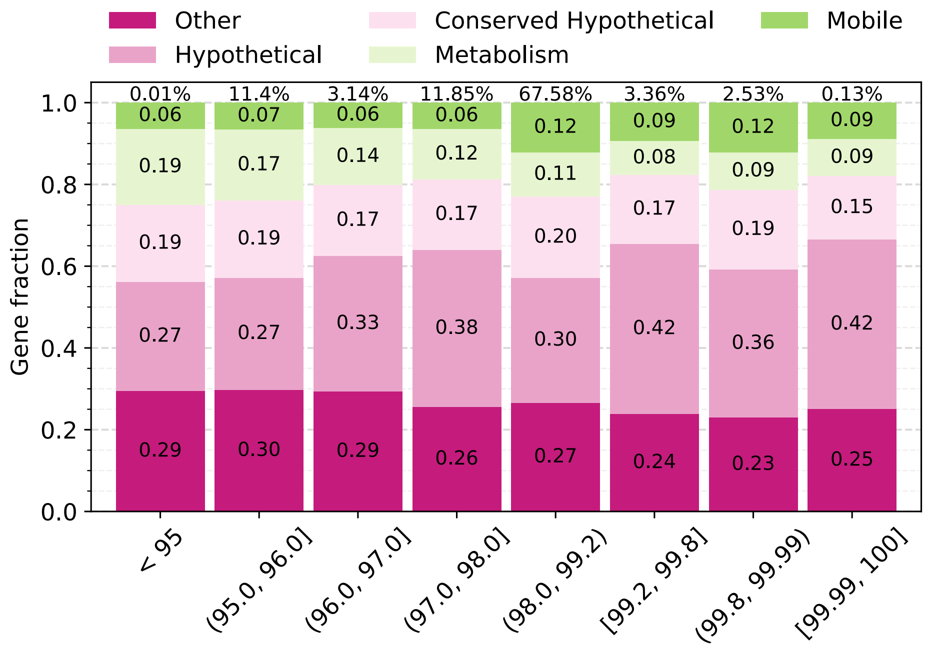

Supplementary Figure S7. Summary statistics of the comparison of the 99.5% ANI threshold to available clonal complexes of *E. coli*. The lines represent the four most abundant STs in terms of number of genomes assigned to them (see key); these STs included ~60% of the total *E. coli* genomes evaluated and are the same as those shown in Fig. 4. F1 statistic (left panel, A and C) and Accuracy (right panel, B and C) were estimated as shown at the bottom of Table S1. Note the increase in accuracy and F1 around 99.5% ANI, consistent with the gap in ANI value distribution in the 99.2-99.8% range.

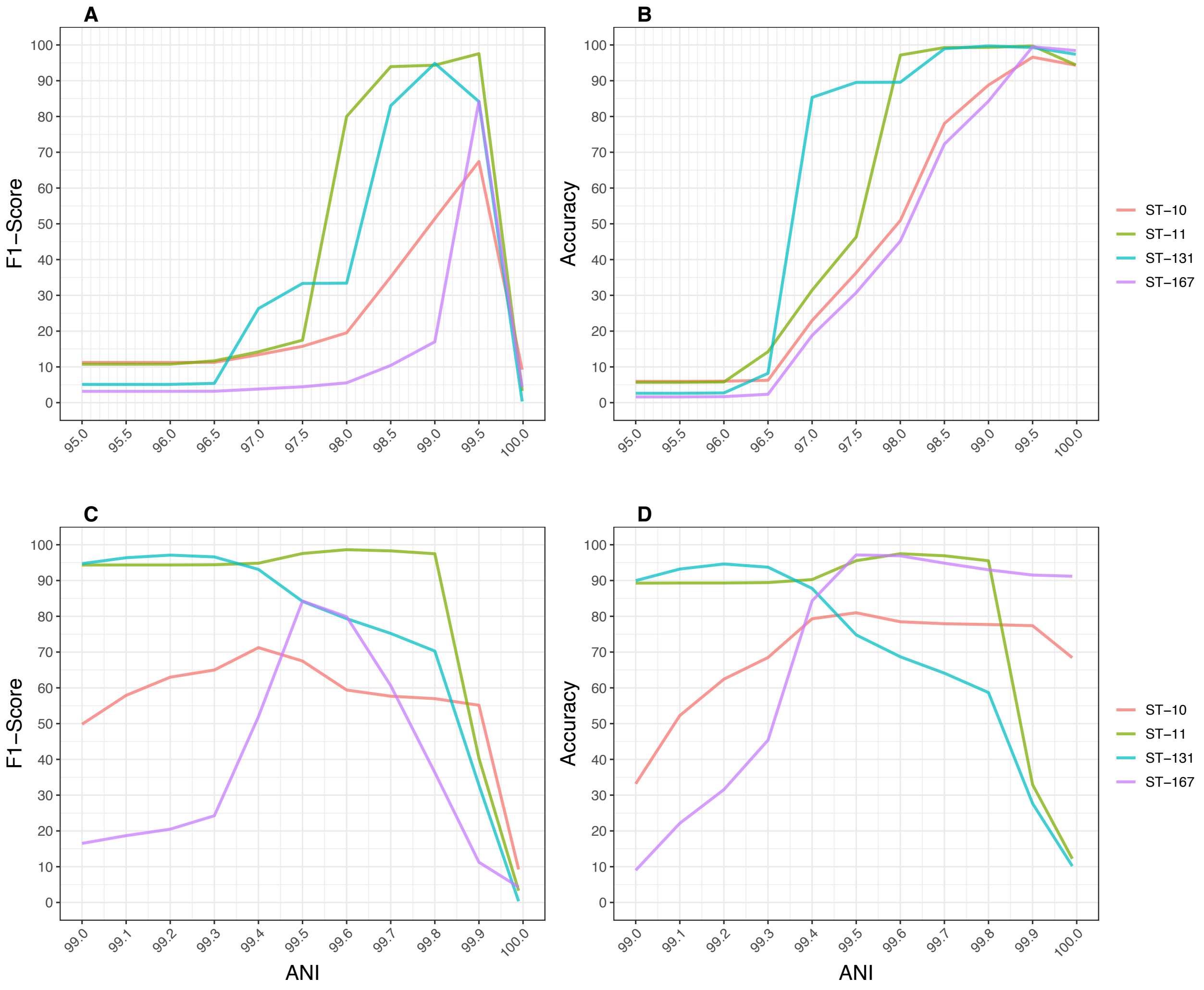

Supplementary Figure S8. Statistical testing of the presence of an ANI distribution gap using the kernel density estimate and bootstrapping. We performed a bootstrap resampling analysis to produce estimates and confidence intervals for local minimum and maximum ranges in the ANI distribution as described in detail in the Supplemental Material and Methods section. Briefly, we filtered our species collection to select only species with at least 100 pairwise genome comparisons ≥ 96.5% ANI leaving 154 species for the bootstrap analysis. Next, for each bootstrap iteration, we randomly sampled with replacement the ANI of 100 pairwise genome comparisons for each species, computed the kernel density estimate across the combined distribution for all 154 species (scipy.stats.gaussian_kde, bw_method=0.15), and identified local minimums and maximums (scipy.signal.find_peaks, default settings). We repeated this for 10,000 bootstrap iterations. The top panel shows the empirical distribution for all species with the local minimum identified at 99.6% ANI. The second panel shows the results of a single bootstrap iteration, and the third panel shows the results from all 10,000 bootstrap iterations (replicates) with the 95% confidence interval surrounding the mean kernel density estimate in blue. The density and spread of local minimum and maximum values are also marked with dark gray or light gray vertical lines in the third panel. The bottom panel shows minimum and maximum results from the third panel as a histogram. Note that the empirical distribution in the top panel is far from a Gaussian or Uniform distribution as there is clear deviation from the mean bin count across the ANI distribution. Note the clearly observed minimum around 99.6% ANI is highly stable and consistent with what is reported in the main text, albeit it is not the only minimum observed (e.g., another minimum is found at 97.8% ANI).

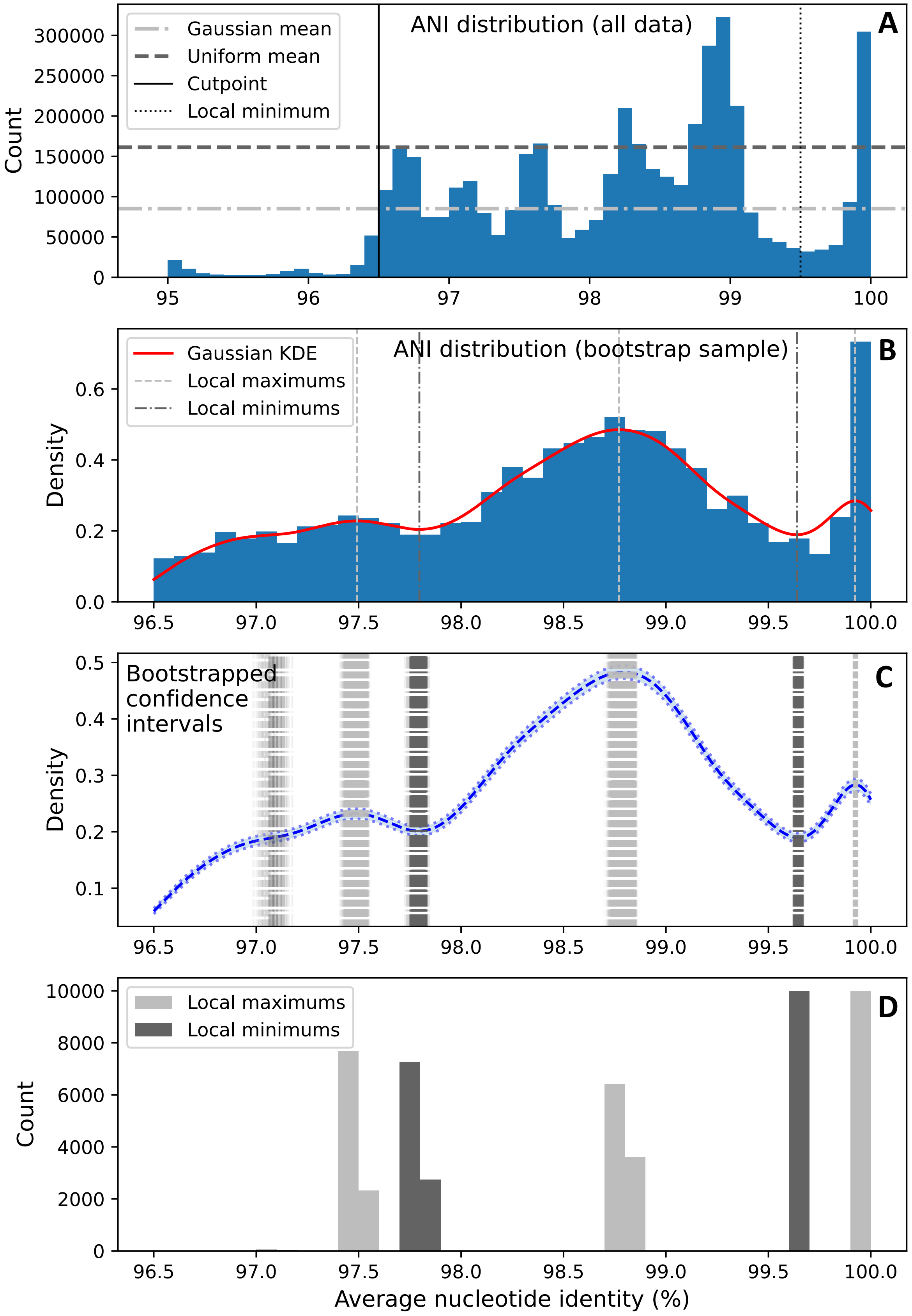

A
ANI distribution (all data)
Gaussian mean
Uniform mean
Cutpoint
Local minimum
Count
300000
250000
200000
150000
100000
50000
0
95
96
97
98
99
100
B
ANI distribution (bootstrap sample)
Gaussian KDE
Local maximums
Local minimums
Density
0.6
0.4
0.2
0.0
96.5
97.0
97.5
98.0
98.5
99.0
99.5
100.0
C
Bootstrapped confidence intervals
Density
0.5
0.4
0.3
0.2
0.1
96.5
97.0
97.5
98.0
98.5
99.0
99.5
100.0
D
Local maximums
Local minimums
Count
10000
8000
6000
4000
2000
0
96.5
97.0
97.5
98.0
98.5
99.0
99.5
100.0
Average nucleotide identity (%)

Supplementary Figure S9. Statistical testing of the presence of an ANI distribution gap using Hartigan's dip test. We produced smooth distributions of ANI in the range between 96 and 100% for each studied species as described in the Supplementary Methods. The pile-up of the resulting distributions revealed a strong gap around 99.6% ANI when including only multimodal distributions (black, upwards from zero), and this gap was also observed between species when including only non-multimodal distributions (gray, downwards from zero; Panel A). Next, we adapted the peak picker algorithm of Weber et al., to the detection of valleys by applying it to the negative of the smoothed densities. The distribution of detected valleys was largely accumulated around the proposed 99.5% ANI value, with a most likely value (mode) estimated at ANI 99.53% (Panel B). Further, ANI values (without smoothing) were classified in one of two Gaussian distributions using model-based clustering, thereby forcing the detection of only the most prominent gap. To characterize the location of the gap, the middle point between the highest ANI from the lower group and the lowest ANI from the higher group was calculated, and set as the cut point between the two Gaussian components. The histogram of cut points, their smoothed density, and most likely value were all estimated as described for valleys above and are shown on Panel C. The most likely value was estimated at 99.555% ANI. Finally, a method for the detection of thresholds derived from image processing was applied to the ANI values, which relies on identifying the threshold that minimizes Otsu's criterion. Again, the histogram, smoothed density, and most likely value of the distribution of detected thresholds were estimated and shown in Panel D. The most likely value was estimated at ANI 99.45%, while values around 99.5% appeared at low frequencies in non-multimodal species, indicating that no effect from false detection was at play. In order to evaluate if any of the above metrics were biased by data size, we compared the squared root of the number of ANI values (roughly corresponding to the total number of genomes considered) with each of the above metrics (Panels E though F). No significant correlations were detected between the size of the data collection for each species and their corresponding values of bimodality (Pearson's R = -0.1, p-value = 0.075) or their detected ANI gaps with any of the methods above ($|R| < 0.053$, p-values > 0.35).

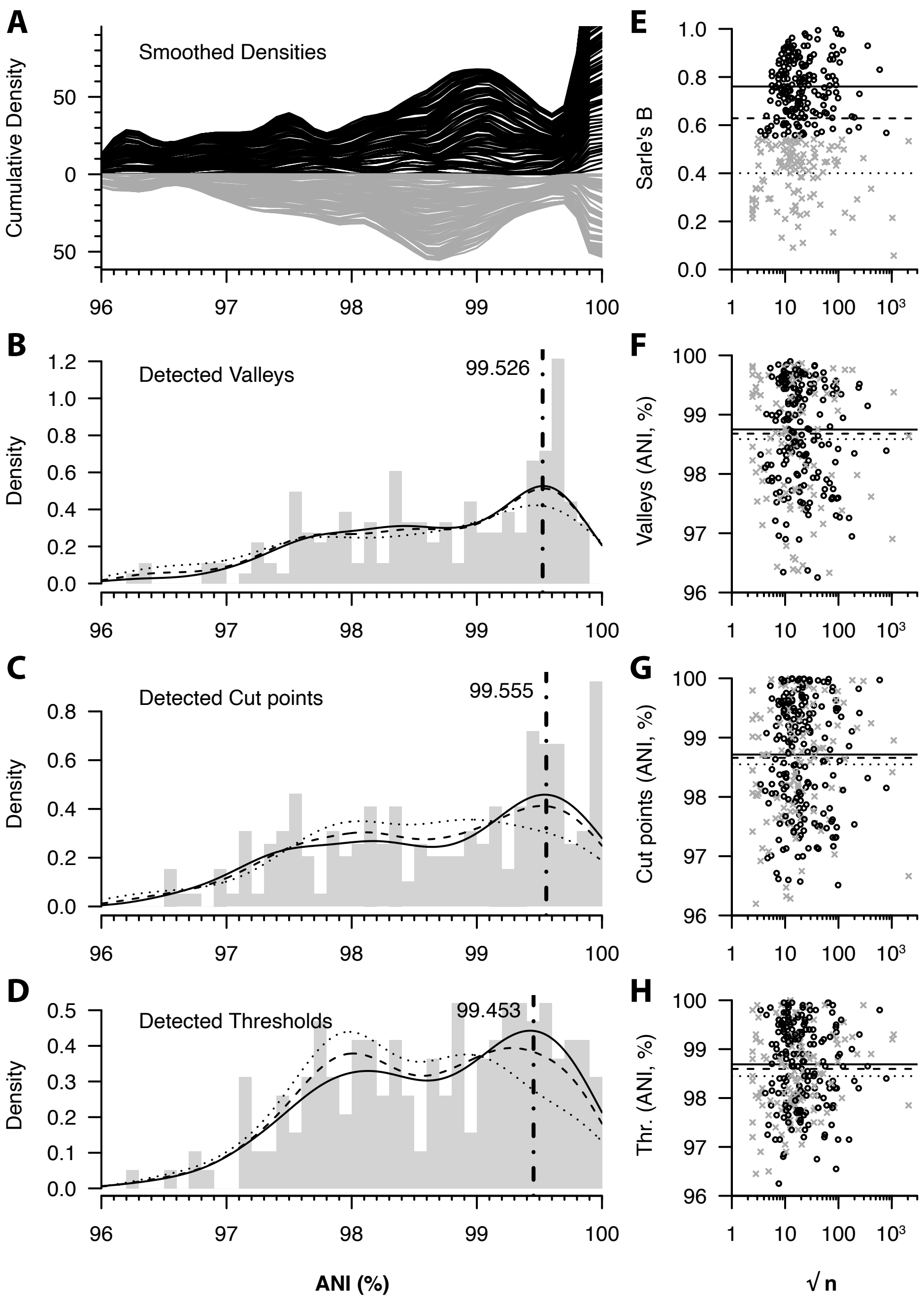

A
Smoothed Densities
Cumulative Density
B
Detected Valleys
99.526
Density
C
Detected Cut points
99.555
Density
D
Detected Thresholds
99.453
Density
ANI (%)
E
Sarle's B
F
Valleys (ANI, %)
G
Cut points (ANI, %)
H
Thr. (ANI, %)
√ n

References and bibliography cited.

1. M. F. Sanner. 1999. Python: a programming language for software integration and development. J Mol Graph Model 17:57-61.

2. C. R. Harris, K. J. Millman, S. J. van der Walt, R. Gommers, P. Virtanen, D. Cournapeau, E. Wieser, J. Taylor, S. Berg, N. J. Smith, R. Kern, M. Picus, S. Hoyer, M. H. van Kerkwijk, M. Brett, A. Haldane, J. F. Del Rio, M. Wiebe, P. Peterson, P. Gerard-Marchant, K. Sheppard, T. Reddy, W. Weckesser, H. Abbasi, C. Gohlke and T. E. Oliphant. 2020. Array programming with NumPy. Nature 585:357-362.

3. P. Virtanen, R. Gommers, T. E. Oliphant, M. Haberland, T. Reddy, D. Cournapeau, E. Burovski, P. Peterson, W. Weckesser, J. Bright, S. J. van der Walt, M. Brett, J. Wilson, K. J. Millman, N. Mayorov, A. R. J. Nelson, E. Jones, R. Kern, E. Larson, C. J. Carey, I. Polat, Y. Feng, E. W. Moore, J. VanderPlas, D. Laxalde, J. Perktold, R. Cimrman, I. Henriksen, E. A. Quintero, C. R. Harris, A. M. Archibald, A. H. Ribeiro, F. Pedregosa, P. van Mulbregt and C. SciPy. 2020. SciPy 1.0: fundamental algorithms for scientific computing in Python. Nat Methods 17:261-272.

4. Y. Okura. 2023. [[Fundamentals] 4. Visualization in Python Programming: How to Use Matplotlib and seaborn]. Nihon Hoshasen Gijutsu Gakkai Zasshi 79:723-731.

5. W. McKinney. 2010. Data structures for statistical computing in python. Proceedings of the 9th Python in Science Conference:51-56.

6. C. De Michele and F. Accatino. 2014. Tree cover bimodality in savannas and forests emerging from the switching between two fire dynamics. PLoS One 9:e91195.

7. J. A. Hartigan and P. M. Hartigan. 1985. The Dip Test of Unimodality. The Annals of Statistics 13:70-84, 15.

8. Y. Benjamini and Y. Hochberg. 1995. Controlling the false discovery rate: a practical and powerful approach to multiple testing. Journal of the Royal Society B 57:289-300.

9. C. M. Weber, S. Ramachandran and S. Henikoff. 2014. Nucleosomes are context-specific, H2A.Z-modulated barriers to RNA polymerase. Mol Cell 53:819-30.

10. L. Scrucca, Fop, M., Murphy , T. B., and Raftery, A. E.,. 2016. mclust 5: Clustering, Classification and Density Estimation Using Gaussian Finite Mixture Models. The R Journal 8:289-317.

11. N. Otsu. 1979. A Threshold Selection Method from Gray-Level Histograms. IEEE Transactions on Systems, Man, and Cybernetics 9:62-66.

12. K. Fukunaga, Hostetler, L. 1975. The estimation of the gradient of a density function, with applications in pattern recognition. IEEE Transactions on Information Theory 21:32-40.

13. K. Pearson. 1916. IX. Mathematical contributions to the theory of evolution.—XIX. Second supplement to a memoir on skew variation. Philosophical Transactions of the Royal Society of London Series A 216:429-457.

14. R. Pfister, K. A. Schwarz, M. Janczyk, R. Dale and J. B. Freeman. 2013. Good things peak in pairs: a note on the bimodality coefficient. Front Psychol 4:700.

15. K. I. V. Chandni S., Meunier C. L., Rick J., Wiltshire K. H., Steinke N., Vidal-Melgosa S., Hehemann J-H., Huettel B., Schweder T., Fuchs B. M., AmannR. I., and Teeling H. 2022. Grazers affect the composition of dissolved storage glycans and thereby bacterioplankton composition during a biphasic North Sea spring algae bloom. BioRxiv doi:https://doi.org/10.1101/2022.09.22.509014:Preprint.
